# Methodology to Promote Physical Activity Monitoring Adherence in Youth with Autism Spectrum Disorder

**DOI:** 10.3389/fpubh.2016.00206

**Published:** 2016-09-27

**Authors:** Janet L. Hauck, Leah R. Ketcheson, Dale A. Ulrich

**Affiliations:** ^1^Physical Activity for Youth with Disabilities (PLAY’D) Laboratory, Department of Kinesiology, Michigan State University, East Lansing, MI, USA; ^2^Center for Physical Activity and Health in Pediatric Disabilities, School of Kinesiology, University of Michigan, Ann Arbor, MI, USA

**Keywords:** accelerometer, measurement, pediatrics, disabilities, pedometer, exercise, obesity, adapted

## Abstract

**Background:**

Objective physical activity (PA) monitoring *via* accelerometry is both costly and time consuming. Furthermore, overall adherence to a monitoring protocol is often complicated by disability. Therefore, it is essential that strategies for supporting accelerometer wear for youth with disabilities are maximized. The purpose of this perspective was to provide researchers a set of efficacious PA monitoring strategies based on the retrospective examination of support methodology on adherence rates for youth with autism spectrum disorder (ASD).

**Method:**

Accelerometer data were collected from 163 participants with ASD in three independent cohorts. Each cohort was provided a varying set of support strategies to help maximize adherence. Chi-square analysis was used to determine differences in adherence between each cohort.

**Results:**

Adherence rates significantly increased from 51.9% in cohort 1 to 88.7% in cohort 2 [χ^2^(1) = 18.333, *p* < 0.001] and again from 88.7% in cohort 2 to 97.4% in cohort 3 [χ^2^(1) = 2.663, *p* = 0.103]. The greatest increase in adherence was observed from 51.9% in cohort 1 to 97.4% in cohort 3 [χ^2^(1) = 19.837, *p* < 0.001]. Support strategies associated with these increases included (1) social story, (2) incentive, (3) concealing techniques, and (4) 24 h/day wear instructions.

**Conclusion:**

Adherence to PA measurement increased when additional support strategies were utilized in combination with a traditional protocol. We recommend these support methodology to be considered as preliminary best practices when measuring objective PA in youth with ASD with likely success in other disability populations.

## Introduction

Autism spectrum disorder (ASD) is the fastest growing developmental disability in the United States (1); as such, there is a critical need to research and intervene on the modifiable factors contributing to known health disparities, such as rising rates of overweight and obesity (2–4). The prevalence of obesity in children with ASD aged 2–19 years is estimated at over 30% (4). One factor which has surfaced as a major health concern contributing to the obesity epidemic is the rise in physical inactivity (5). Evidence suggests that both typically developing children and children with disabilities are falling well below recommended physical activity (PA) guidelines with increasing sedentary behavior as they age (2, 6–10). In addition, given the current spot light on motor skill competence in children with ASD, motor skill interventions focused on motor and PA outcomes are gaining significant attention yielding the need for improved measurement methodology (8, 11–20). In order to maintain insight of the levels and patterns of PA in youth, valid and reliable methods of objective PA measurement must be incorporated. Therefore, it is imperative to continue to evolve evidence-based best practices in activity measurement to enhance research efforts in understanding and addressing this global epidemic.

Current best practices in activity measurement include an objective measurement of PA using motion detectors such as an accelerometer which is worn around the waist, wrist, or ankle (21). Despite recent reports highlighting the importance of measuring PA using an objective method (21), many researchers still resort to implement self-reported and recall instruments. Common methods include retrospective measurement of previous activity with recall varying from 24 h to 1 year, a parental report of the child’s activity, or an activity interview with a log (22). There are several issues with self-reported measures, including inaccurate representation on the frequency, duration, and intensity of PA following bouts of activity which are retrospectively recalled (22). Additionally, youth and their caregivers have a tendency to over-report their PA (21). Despite these issues, self-reported questionnaires are often considered for cost efficiency reasons. Caution, however, should be taken when considering results from large epidemiological studies, with outcomes associated with programing or intervention recommendations when recall methodology is used. To avoid misrepresenting the levels of PA in youth, particularly those most sensitive to program modifications (children with disabilities), employing an objective measurement such as accelerometry, can more accurately inform policy makers and service providers regarding the current PA behavior of targeted populations.

An important component used to inform PA measurement methodology for future research should be the reporting of adherence results from prior studies. Unfortunately, reporting of adherence to PA measurement procedures is rarely published (7, 23, 24). Adherence to accelerometer measurement procedures is likely to vary by population (children or adults) and experience (no. of observations). However, reporting the number of participants who are able to successfully achieve the minimum amount of wear needed to meet reliability criteria provides an accurate estimate of participants who may potentially adhere in future studies; valuable information which can be used to inform sample size and research budgets. Similarly, studies examining the minimum days and hours of PA monitoring required for reliability have the potential to reduce participant burden while increasing adherence (25, 26). A recent study examined the minimum number of days required to reliably measure PA in youth with developmental disabilities (27). Results indicated that 4, 6, and 8 days of monitoring were required to reliably quantify typical levels of PA during the week, weekend, and combined week and weekend, respectively (27). Additional studies examining the minimum number of days required for monitoring will provide further support for these findings, and ultimately provide valuable information which can be used to develop best practices when examining PA in youth with disabilities.

Another consideration in the measurement of objective PA *via* accelerometry in youth is cost. The majority of expenses are typically associated with cost of the device, which can range from $250 to $450. Required software costs also vary substantially. In addition to hardware and software costs, expenses relating to research procedures can accumulate. One such cost is providing incentive. Since PA monitoring can be burdensome for some children, particularly those with disabilities, it may be wise to provide an incentive for wearing the monitor; a recommendation that is substantiated by the current report. Also, the cost of postage should be considered given that monitors are most efficiently delivered and returned *via* mail. Finally, data reduction and analysis are time consuming; therefore, it is also wise to consider the cost of hiring personnel to assist with this process. Despite these considerable expenses which are further burdened by poor adherence, the reporting of strategies to increase PA monitoring adherence is scarce.

Taken together, objective PA monitoring *via* accelerometry is both costly and time consuming. Therefore, it is essential that strategies for supporting accelerometer wear for youth with disabilities are maximized (24). The purpose of this retrospective investigation was to compare adherence rates of accelerometer measurement protocols across three independent cohorts, to differentiate the effects of support strategies offered to increase protocol adherence, and to offer our perspective on accelerometer adherence promoting strategies that should be considered when monitoring PA in children and youth ASD.

## Method

### Participants

We performed a retrospective comparison of PA monitoring adherence data obtained from three independent cohorts (28–30). For each cohort, an independent sample was recruited to participate in an intervention which included the measurement of habitual PA *via* an accelerometer. A total of 163 youth aged 9–18 years with ASD participated (cohort 1, *n* = 27; cohort 2, *n* = 97; cohort 3, *n* = 39). Demographic data for each cohort are summarized in Table 1. The research protocol was approved by the Health Sciences and Behavioral Sciences Institutional Review Board. All participants were consented to participate and assent was obtained from each.

**Table 1:** **Demographic data of participants by cohort**.

Characteristics	Cohort 1 *n* = 27	Cohort 2 *n* = 97	Cohort 3 *n* = 39
% of female	27	23	19
Age (years)	11.9 ± 2.4	12.1 ± 2.3	11.8 ± 2.5
Height (cm)	147.0 ± 12.9	149.6 ± 13.3	145.1 ± 10.9
Weight (kg)	46.9 ± 19.3	48.8 ± 19.9	40.4 ± 14.3
BMI	20.6 ± 7.0	21.1 ± 5.4	18.8 ± 4.3
SRS score	–	81.0 ± 13.9	81.8 ± 13.0
ADOS-2 CSS	–	–	6.75 ± 1.5
WASI-2	–	81.0 ± 18.9	85.5 ± 19.7
PPVT-4	74.9 ± 26.4	–	–

*BMI, Body Mass Index; SRS, Social Responsiveness Scale; ADOS, Autism Diagnostic Observation Schedule; CSS, Calibrated Severity Score; WASI, Wechsler Abbreviated Scale of Intelligence; PPVT, Peabody Picture Vocabulary Test*.

### Procedure

A baseline measure of objective PA *via* an accelerometer was collected for each cohort. Type of accelerometer used was individually decided by the research team for each cohort and therefore varies. The Actical accelerometer (Actical, Philips Respironics; Bend, OR, USA) was used for cohorts 1 and 2. The Actigraph accelerometer (Actigraph GT3X, Actigraph; Pensacola, FL, USA) was used for cohort 3. These devices are very similar in size and shape and therefore are not considered as a factor affecting adherence.

### Data Reduction

Data were reduced using specific reliability criteria and validated cut points (31, 32). Data were considered reliable when the accelerometer was worn for a minimum of 10 h on 4 days in short succession, one of which must have been a weekend day. Data were only included in further analysis if reliability criteria were met. We used reliability criteria as the critical factor in determining whether adherence was met. If criteria were met, then adherence to the PA protocol was met, yielding usable and reliable data. If criteria were not met, then adherence was also not met.

### Adherence Methodology

For each cohort, a standardized set of adherence strategies were provided to participants in an effort to increase the likelihood that reliability criteria were met. With each successive cohort, new adherence strategies were added to the original protocol procedures at the discretion of the research team. Original protocol procedures used consistently for all three cohorts included a 1-week monitoring period, providing written instructions, a monitoring log, and providing a self-addressed and stamped envelope for returning the monitor. Additional adherence strategies implemented for cohorts 2 and 3 included verbal instructions, in-person training, providing a social story, increased daily wear instructions to 24 h/day, concealing and decorating wear techniques, and incentive payment (cohort 3 only). Due to the *post hoc* nature of this investigation, we were unable to manipulate support strategies among the three samples in a systematic way, limiting specific interpretations. Please see Table 2 for a full description of protocol procedures and adherence strategies as well as which cohorts incorporated their use.

**Table 2:** **Use of traditional accelerometry procedures and additional support strategies by cohort**.

Adherence supports^a^	Brief description	Cohort 1 *n* = 27	Cohort 2 *n* = 97	Cohort 3 *n* = 39
Monitoring period^b^	Amount of time participants are instructed to wear accelerometer	1 week	1 week	1 week
Method of delivery				
Mail	Participants received accelerometer by mail	x		x
In person	Participants received accelerometer in person		x	
Instructions				
Written^b^	Provided a simple letter detailing instructions	x	x	x
Verbal	Provided in-person verbal instructions		x	
In-person training	Conducted home visits to provide in-person training		x	
Social story	Provided social story to increase comprehension and reduce anxiety		x	x
Monitor log^b^	Provided a monitoring log to record non-wear periods	x	x	x
Wear time instructions				
Waking hours	Participants instructed to wear accelerometer during waking hours	x		
24 h/day	Participants instructed to wear accelerometer 24 h/day		x	x
Wear techniques				
Concealing	Taught participants to conceal accelerometer under clothing		x	x
Decorating	Allowed participants to decorate accelerometer with stickers		x	
Returned by mail^b^	Participants returned accelerometer in pre addressed/stamped envelop	x	x	x
Incentive	Incentive provided upon return of accelerometer with complete data			x

*x = used adherence support*.

*^a^Supports are presented as a semi-sequential timeline of when supports are typically utilized during physical activity measurement*.

*^b^Original protocol procedures*.

### Statistical Analysis

All PA data were reduced with computer software using the previously mentioned reliability criteria. Actical data were reduced using a specially designed computer program (33). Actigraph data were reduced using ActiLife 6.0. Adherence was used as a binary outcome variable and classification of “adherence” or “non-adherence” was based on whether or not reliability criteria were met (10 h/4 days + 1 weekend day). Analyses included determining the percentage of the sample that adhered to the PA measurement protocol for each study. Chi-square analysis was used to determine differences in adherence between each cohort. Support strategies utilized were then compared and contrasted accounting for observed differences in adherence outcomes.

All statistical analyses were conducted using IBM SPSS Statistics 20 (SPSS Inc., Chicago, IL, USA). Differences were considered significant at an alpha level of 0.05.

## Results

The results present *post hoc* comparison of accelerometry adherence rates from three independent cohorts, each of which incorporated a varying combination of support methodology. For cohort 1, 14 of 27 participants met reliability criteria indicating 51.9% adherence. For cohort 2, 86 of 97 participants met reliability criteria indicating 88.7% adherence. For cohort 3, 38 of 39 participants met reliability criteria indicating 97.4% adherence.

To evaluate the meaningfulness of additional adherence support strategies from one cohort to another, group differences of adherence were calculated using chi-square. Adherence significantly increased between cohorts 1 and 2 [χ^2^(1) = 18.333, *p* < 0.001]. Additional supports included (1) in-person delivery of accelerometer, (2) verbal instructions, (3) in-person training, (4) providing a social story, (5) increased daily wear instructions to 24 h/day, (6) concealing wear techniques, and (7) decorating wear techniques.

There was a non-significant increase in adherence between cohorts 2 and 3 [χ^2^(1) = 2.663, *p* = 0.103]. One additional support was included during that period. Incentive payment was provided to participants upon the return of the accelerometer containing complete data. Supports that were omitted for cohort 3 which had been previously utilized for cohort 2 included (1) in-person delivery of accelerometer, (2) verbal instructions, (3) in-person training, and (4) decorating wear techniques.

Adherence significantly increased between cohorts 1 and 3 [χ^2^(1) = 19.837, *p* < 0.001]. Additional supports included (1) providing a social story, (2) increased daily wear instructions to 24 h/day, (3) concealing wear techniques, and (4) providing incentive payment.

## Discussion

Successful adherence to objective PA measurement protocol for youth with ASD aged 9–18 years was increased over the progression of three independent cohorts. This improvement coincides with the addition of many adherence support strategies. The contribution of each subsequent support is suspected of influencing the significant increase observed in adherence. These results are positive and the first to contribute to the development of best practices when conducting objective PA measurement *via* an accelerometer in youth with disabilities.

Adherence rates significantly increased by 45.5% from cohort 1 to cohort 3. Adherence support strategies that both cohorts 2 and 3 shared in common include providing a social story, increasing wear time to 24 h/day, and offering concealing tips to participants. These supports were not included in the first cohort. Given the progression of increased adherence, it is likely that the addition of these supports to the PA measurement protocol influenced adherence. However, caution must be warned in making an assumption of causation given the lack of a true control group.

To further facilitate improved adherence, one additional support was utilized for cohort 3 which had not been previously attempted. Participants were offered a small monetary incentive if they returned the accelerometer with complete data, meaning that they wore the monitor long enough to accumulate the minimum amount of data to achieve reliability. The use of the incentive support stimulated an additional non-significant increase in adherence of 8.7% between cohorts 2 and 3, even in the absence of other helpful supports utilized for cohort 2. Anecdotal evidence suggested that providing the incentive gave participants (or their caregivers) a perceived benefit in completing the PA measurement, thereby increasing dedication to the task. In addition, the research garnished a higher return rate of monitors when incentive was offered.

We believe that the incentive support greatly improved adherence, to the extent that we are now tentative in recommending other supports that were only offered to cohort 2. Those supports included in-person delivery of the accelerometer with in-person training and verbal instructions. These supports clearly increased adherence by 36.8% between cohorts 1 and 2; however, we demonstrated that adherence continued to improve even after omitting these supports for cohort 3 when an incentive is offered. Given the increased costs to the research in time, effort, and budget, we do not recommend in-person delivery, in-person training, or verbal instructions be incorporated into your measurement protocol unless resources allow for these extra costs, so long as incentive is offered following the return of the accelerometer with complete data.

Limitations exist which could affect interpretations. First and foremost, we are not examining the effects of sample characteristics to adherence of accelerometer procedures, but rather the effects of the adherence supports and instructional procedures provided by the research team. Assessments utilized to summarize IQ and ASD severity varied between cohorts, essentially limiting such analyses. Despite this, IQ determined *via* the Peabody Picture Vocabulary Test-4 and the Wechsler Abbreviated Scale of Intelligence-2 resulted in comparable IQs for all cohorts. Minimal differences in BMI are also noted, with no statistically significant differences between cohorts. Despite this, the relation of adherence rates to BMI was not investigated. Additionally, we are not comparing PA between the samples. For that reason, information regarding epoch length, cut points, and PA intensities are not reported. Finally, this retrospective data analysis was performed after the conclusion of measurement of all cohorts, yielding us little control over the systematic planning of support use, accelerometer type, or population. Having said this, the research made every effort to control for environmental and participant demographics. Measurement occurred during a 1-week period in the spring time in the same geographical region in the United States during each study. The sample included only youth diagnosed with ASD. The mean age of participants across studies was stable, only varying by a few months. Accelerometer model varied, but most would conclude this had little impact on adherence given the size similarities between the Actical and Actigraph.

These limitations prompt caution regarding causation. Given the lack of a true control group or the inability to manipulate support use in a systematic way, it is difficult to quantify the individual effects of each strategy. Also, we are unable to differentiate between child and parent driven adherence but can attest that all supports were provided to parents by the research team and in many cases were disseminated to the child from the parent. Finally, we acknowledge our limited ability to remark on the influence of autism severity and functional status on adherence rates. Given inherent variability in the severity and functional status of children on the autism spectrum, a well-controlled prospective study is warranted to validate the effectiveness of these strategies for enhancing PA monitoring adherence in children with comparable functioning.

As a result of incorporating multiple adherence supports into the practice of objective PA measurement *via* an accelerometer for youth with ASD, adherence rates increased yielding more reliable data and fewer missing data. One common issue experienced by PA researchers are participants who wear the device as described but fall just short of reaching the minimum amount of wear time needed to meet reliability criteria. Oftentimes, these participants’ miss reliability criteria by only a few hours on a given day or by missing a weekend day of wear. For this reason, it is important that research continue to examine the minimum wear criteria needed to produce reliable data in special populations. If criteria are reduced, adherence will likely be increased. A reduction of wear time would decrease research costs and ease burden for participants.

In conclusion, adherence to objective PA measurement *via* an accelerometer can be increased when extra support strategies are utilized. We feel that adherence is stimulated most by increasing wear time to 24 h/day, providing a social story, offering concealing tips to participants, and providing an incentive upon the return of the accelerometer with complete data. These support strategies should be used in combination with traditional protocol components including a 1-week monitoring period, providing written instructions, a monitoring log, and providing a self-addressed stamped return envelope. These methods were recently utilized with a younger sample of children with ASD with a 95% adherence rate (11, 34). This suggests utility of these supports in younger samples. These strategies can be generalized to objective PA monitoring using other devices such as pedometers and for use in children without disabilities. Adding these strategies to your pediatric PA measurement protocol is likely to increase research efficiency and decrease non-incentive related costs. Finally, we advise these support methodology be strongly considered as preliminary best practices when measuring objective PA in youth with ASD.

## Author Contributions

JH contributed to the research design, data collection, statistical analysis, and preparation of the draft of the manuscript. LK contributed to data collection, statistical analysis, and editing the draft of the manuscript. DU contributed to the research design, securing funds, data collection, and editing the draft of the manuscript.

## Conflict of Interest Statement

The authors declare that the research was conducted in the absence of any commercial or financial relationships that could be construed as a potential conflict of interest.
